# [^18^F]FDG uptake of axillary lymph nodes after COVID-19 vaccination in oncological PET/CT: frequency, intensity, and potential clinical impact

**DOI:** 10.1007/s00330-021-08122-2

**Published:** 2021-06-22

**Authors:** Stephan Skawran, Antonio G. Gennari, Manuel Dittli, Valerie Treyer, Urs J. Muehlematter, Alexander Maurer, Irene A. Burger, Cäcilia Mader, Olivia Messerli, Hannes Grünig, Catherine Gebhard, Martin W. Huellner, Alessandra Curioni-Fontecedro, Christoph Berger, Michael Messerli

**Affiliations:** 1 Department of Nuclear Medicine, University Hospital Zurich, Rämistrasse 100, CH-8091 Zurich, Switzerland; 2 University of Zurich, Zurich, Switzerland; 3 Department of Nuclear Medicine, Kantonsspital Baden, Baden, Switzerland; 4 Department of Dermatology, University Hospital Zurich, Zürich, Switzerland; 5 Department of Medical Oncology and Hematology, University Hospital Zurich, Zurich, Switzerland; 6 Division of Infectious Diseases and Children’s Research Centre, University Children’s Hospital Zurich, Zurich, Switzerland

**Keywords:** COVID-19 vaccines, Lymph nodes, Fluorodeoxyglucose F18, Positron-emission tomography

## Abstract

**Objectives:**

To assess the frequency, intensity, and clinical impact of [^18^F]FDG-avidity of axillary lymph nodes after vaccination with COVID-19 vaccines BNT162b2 (Pfizer-BioNTech) and mRNA-1273 (Moderna) in patients referred for oncological FDG PET/CT.

**Methods:**

One hundred forty patients referred for FDG PET/CT during February and March 2021 after first or second vaccination with Pfizer-BioNTech or Moderna were retrospectively included. FDG-avidity of ipsilateral axillary lymph nodes was measured and compared. Assuming no knowledge of prior vaccination, metastatic risk was analyzed by two readers and the clinical impact was evaluated.

**Results:**

FDG PET/CT showed FDG-avid lymph nodes ipsilateral to the vaccine injection in 75/140 (54%) patients with a mean SUV_max_ of 5.1 (range 2.0 – 17.3). FDG-avid lymph nodes were more frequent in patients vaccinated with Moderna than Pfizer-BioNTech (36/50 [72%] vs. 39/90 [43%] cases, *p *< 0.001). Metastatic risk of unilateral FDG-avid axillary lymph nodes was rated unlikely in 52/140 (37%), potential in 15/140 (11%), and likely in 8/140 (6%) cases. Clinical management was affected in 17/140 (12%) cases.

**Conclusions:**

FDG-avid axillary lymph nodes are common after COVID-19 vaccination. The avidity of lymph nodes is more frequent in Moderna compared to that in Pfizer-BioNTech vaccines. To avoid relatively frequent clinical dilemmas, we recommend carefully taking the history for prior vaccination in patients undergoing FDG PET/CT and administering the vaccine contralateral to primary cancer.

**Key Points:**

*• PET/CT showed FDG-avid axillary lymph nodes ipsilateral to the vaccine injection site in 54% of 140 oncological patients after COVID-19 vaccination.*

*• FDG-avid lymphadenopathy was observed significantly more frequently in Moderna compared to patients receiving Pfizer-BioNTech-vaccines.*

*• Patients should be screened for prior COVID-19 vaccination before undergoing PET/CT to enable individually tailored recommendations for clinical management.*

## Introduction

The COVID-19 virus pandemic has deeply affected worldwide healthcare systems. To this day, the detrimental effect the virus exerts on the respiratory system is a major concern for global health, particularly in smokers, elderly, or patients with malignant tumors [1]. Among the latter, the fatality rate was higher in infected patients, even after adjusting for confounders [2]. Therefore, cancer patients were prioritized to receive BNT162b2 (Pfizer-BioNTech) and mRNA-1273 (Moderna) vaccines.

Pain at the site of injection site and ipsilateral axillary lymphadenopathy were acknowledged as COVID-19 vaccines’ side effects [3, 4]. Similar findings have been reported with other vaccines (e.g., H1N1, papillomavirus) on imaging studies [5]. Cases of cancer patients with metabolically active axillary lymph nodes are burgeoning in the literature after F18-fluorodeoxyglucose ([^18^F]FDG) positron emission tomography/computed tomography (PET/CT) [6], sometimes leading to unnecessary lymph node core needle biopsy [6, 7]. Different expert consensus opinions for the management of axillary adenopathy in patients with recent COVID-19 vaccination undergoing imaging (e.g., from the Society of Breast Imaging) were recently published [8]. A very recent publication from Israel with 728 vaccinated patients reported an increased rate of FDG-avid axillary lymph nodes in 46% of patients undergoing PET/CT after vaccination with Pfizer-BioNTech [9]. Further evidence is sparse with only case reports or small cohort studies published and in-depth knowledge about the impact of prior vaccination on PET imaging, especially using other vaccines than Pfizer-BioNTech is currently lacking.

Accordingly, the aims of this study were to analyze the overall frequency and intensity of [^18^F]FDG PET/CT avid axillary lymph nodes ipsilateral to COVID-19 vaccination injection site, to compare Pfizer-BioNTech and Moderna vaccines’ reactogenicity, and to assess potential clinical impact.

## Materials and methods

### Study design and population

In this retrospective cross-sectional single-center study, we included all patients who underwent a clinically indicated [^18^F]FDG-PET/CT after vaccination with either BNT162b2 (Comirnaty®, Pfizer/BioNTech) or mRNA-1273 (Moderna®, Moderna Biotech) during the study period from 2^nd^ of February 2021 to 19^th^ of March 2021. Clinical information including age, sex, body mass index (BMI), and oncological diagnosis was recorded (Table 1). Dates of the first and, if applicable, second vaccination as well as the vaccine administered were recorded. Written informed consent for the scientific use of medical data was obtained from all patients. The study was approved by the local ethics committee and was conducted in compliance with ICH-GCP rules and the Declaration of Helsinki.

**Table 1:** Demographic data of study subjects (*n* = 140)

Female/male, *n* (%)	39 (28%)/101 (72%)
Age, years	67 ± 13 (25–94)
Body weight, kg	75 ± 17 (40–137)
Body height, m	1.73 ± 0.09 (1.48–1.93)
BMI, kg/m^2^	24.9 ± 4.8 (15.8–45.8)
Blood glucose level at time of injection, mg/dL	105 ± 19 (67–203)
Injected FDG activity, MBq	201 ± 63 (86–335)
PET/CT scan post injection time, min	60 ± 7 (46–89)
Time interval between vaccination and PET/CT, days	17 ± 11 (0–48)
Type of primary disease	
Melanoma and other skin cancers	45 (32%)
Lung and mediastinal tumors	35 (25%)
Lymphoma	16 (11%)
Head and neck cancer	14 (10%)
Paraneoplastic syndrome	6 (4%)
Colorectal cancer	5 (4%)
Breast cancer	4 (3%)
Pancreatic cancer	4 (3%)
Cholangiocarcinoma	4 (3%)
Mesothelioma	2 (1%)
Urogenital cancer	2 (1%)
Cancer of unknown origin	2 (1%)
Esophageal cancer	1 (1%)

Values are given as absolute numbers and percentages in parenthesis or mean ± standard deviation (range)

*BMI*, body mass index; *MBq*, Mega-Becquerel; *PET/CT*, positron emission tomography/computed tomography

### PET acquisition and image reconstruction

Examinations were performed on a latest-generation PET/CT scanner (GE Discovery MI, GE Healthcare) using a standardized clinical protocol with a BMI-adapted [^18^F]FDG dosage protocol as described in detail [10]. Participants fasted for at least 4 h prior to the [^18^F]FDG tracer injection. The [^18^F]FDG uptake time was set to 60 min. A CT scan was obtained from the vertex of the skull to the mid-thighs or feet (e.g., in case of lower extremity melanoma) and used for anatomical localization of [^18^F]FDG uptake as well as attenuation correction. The CT scan was acquired using automated tube dose modulation (range 15–100 mA) with 120 kV. Following the CT acquisition, the PET images were acquired covering the identical anatomical region. The PET acquisition time was 2 min per bed position, with 6–11 bed positions per patient (depending on patient size), using an overlap of 23% (17 slices). The PET was acquired in 3D mode and slice thickness was 2.79 mm. PET reconstructions were generated using penalized likelihood reconstruction (Q.Clear, GE Healthcare) with a *β*-value of 450. All PET datasets were reconstructed with a 256 × 256 pixel matrix.

### [^18^F]FDG PET/CT data analysis

One reader (A.G.G., 6 years of experience in radiology) reviewed all PET data sets. Commercial image analysis software (Advantage Workstation Version 4.7, GE Healthcare) was used for the review. The reader measured FDG avidity by drawing a semi-automated cubicle volume of interest (VOI) around the most avid axillary lymph nodes bilaterally. FDG avidity was measured as the maximum standardized uptake value (SUV_max_) within the VOI (i.e., decay corrected radioactivity per volume [kBq/mL], divided by the initially injected activity [MBq] and multiplied by body weight [kg]). The absolute difference of SUV_max_ between the data pairs of axillary lymph nodes was calculated. A positive reaction was defined as unilateral FDG-avidity of axillary lymph nodes ipsilateral to the prior vaccination being *(a)* at least one visually depicted lymph node on maximum intensity projection (MIP), and *(b)* having a difference in SUV_max_ > 0.5 (avidity ipsilateral lymph nodes − avidity contralateral lymph node). Only cases with positive reactions were included in quantitative analyses of avidity. Two patients were excluded because of extensive bilateral axillary FDG-uptake in the lymph nodes due to active lymphoma and the fact that bilaterally FDG-avid lymph nodes are very unlikely after (unilateral) vaccination.

### Assessment of metastatic risk and clinical impact of [^18^F]FDG avid lymph nodes

In a second reading session, two readers (S.S., 5 years of experience in radiology and M.M., 9 years of experience in radiology and 3 years in PET reading and board-certified in radiology and nuclear medicine) reviewed all cases with FDG-avid lymphadenopathy to assess metastatic risk. For that purpose, the readers had access to clinical information on primary tumor/primary oncological malignancy and other clinical information (e.g., primary stage, previous pathology findings). Metastatic risk was assessed as follows: In a patient without a history of previous vaccination based on localization and intensity of FDG-avid axillary lymphadenopathy, this finding would be *(1) unlikely* to represent metastasis, e.g., abdominal primary without infradiaphragmal metastasis; *(2) potentially* represent metastasis, e.g., lymphoma with active axillary lymph nodes; *(3) likely* to represent metastasis, e.g., breast cancer with ipsilateral [^18^F]FDG avid lymph nodes. Furthermore, on a per-patient basis, the impact on clinical management was as follows: In consensus with a board-certified medical oncologist (A.C.F., 11 years of experience in oncology), cases with [^18^F]FDG avid lymph nodes were reviewed, simulating a scenario without knowledge of previous vaccination. Cases, where the finding would have resulted in change of clinical management, were recorded and recommendations formulated accordingly.

### Statistical analysis

All statistical analyses were performed in the open-source statistics software R (version 3.6.1, R Foundation for Statistical Computing) [11]. Categorical variables are expressed as frequency distribution. Continuous variables are presented as mean ± standard deviation if normally distributed or median (range) otherwise. Assessment of group differences was determined using an unpaired *t*-test after ensuring a normal distribution of the data using the Shapiro-Wilk test. For non-normally distributed data, a Wilcoxon-test or chi-square-test was used. F18-FDG avidity of axillary lymph nodes was plotted over time interval after vaccination and a locally weighted scatterplot smoothing curve was fitted using R. Spearman’s coefficient of rank correlation was calculated. For all comparisons, a *p* value of < 0.05 was considered to be statistically significant.

## Results

### Patient characteristics

Demographic data of the study patients (*n* = 140) is given in Table 1. One hundred forty patients that were vaccinated with COVID-19 vaccines prior to undergoing [^18^F]FDG PET/CT for re-/staging of oncological diseases were retrospectively included in the study. The average BMI was 24.9 ± 4.8 (range 15.8–45.8) kg/m^2^ and the mean injected [^18^F]FDG activity was 200.9 ± 62.7 (range 85.7–334.7) MBq. Ninety patients (64%) were vaccinated with Pfizer-BioNTech and 50 patients (36%) were vaccinated with Moderna vaccines. Forty-eight patients (34%) were vaccinated for the first time and 92 patients (66%) received the second vaccination. The mean time interval between vaccination and [^18^F]FDG PET/CT scanning was 17 ± 11 (range 0–48) days. Underlying etiologies most frequently were melanoma (45/140 patients), lung and mediastinal tumors (35/140 patients), lymphoma (16/140 patients), and head and neck cancer (14/140 patients) with the remaining explicitly given in Table 1.

### FDG-avidity of the axillary lymph nodes ipsilateral to COVID-19 vaccination

Overall, the average SUV_max_ of axillary lymph nodes ipsilateral to the injection was 3.3 ± 3.0 (range 0.3–17.3). There were 75/140 (54%) patients presenting with [^18^F]FDG-avid lymph nodes ipsilateral to the vaccine injection with an average SUV_max_ 5.1 ± 2.1 (range 2.0 to 17.3). The frequencies of [^18^F]FDG-avid lymph nodes after vaccination discretized to the time delay in weeks are given in Table 2. During the first 7 days after vaccination, 22/31 (71%) patients presented with FDG-avid lymph nodes (Pfizer-BioNTech, *n* = 18; Moderna, *n* = 4). After 28 days post vaccination, 9/24 (38%) patients (Pfizer-BioNTech, *n* = 7; Moderna, *n* = 2) presented with unilateral FDG-uptake in the axillary lymph nodes. Spearman’s coefficient of rank correlation showed a weak correlation between FDG-uptake and days after vaccination (rho = −0.268; *p* value = 0.001).

**Table 2:** Patients with reaction to COVID-19 vaccination in regard to time delay between vaccination and PET/CT scan

			Increased [^18^F]FDG uptake in axillary lymph node		
	Total patients	SUV_max_	Yes	No	SUV_max_a
Overall	140 (100%)	3.3 ± 3.0 (0.3–17.3)	75 (54%)	65 (46%)	5.1 ± 2.1 (2.0–17.3)
Days 0–7	31 (22%)	5.2 ± 4.3 (0.3–17.3)	22 (71%)	9 (29%)	6.8 ± 4.1 (2.0–17.3)
Days 8–14	33 (24%)	3.3 ± 2.8 (0.4–10.1)	17 (52%)	16 (48%)	5.2 ± 2.8 (2.2–10.1)
Days 15–21	35 (25%)	2.8 ± 2.1 (0.4–9.5)	18 (51%)	17 (49%)	4.3 ± 1.9 (2.2–9.5)
Days 22–28	17 (12%)	2.6 ± 2.0 (0.7–8.2)	9 (53%)	8 (47%)	3.8 ± 2.0 (2.0–8.2)
Day > 28	24 (17%)	2.1 ± 1.6 (0.4–6.8)	9 (38%)	15 (62%)	3.9 ± 1.4 (2.3–6.8)

Values are given as absolute numbers and percentages in parenthesis or mean ± standard deviation (range)

*PET/CT*, positron emission tomography/computed tomography; *SUV*_*max*_, maximum standardized uptake value

^a^i.e., SUV_max_ of all patients (*n* = 75) with increased FDG uptake

### Differences of FDG-avidity according to vaccines used and 1. vs. 2. vaccination

**Fig. 1:**
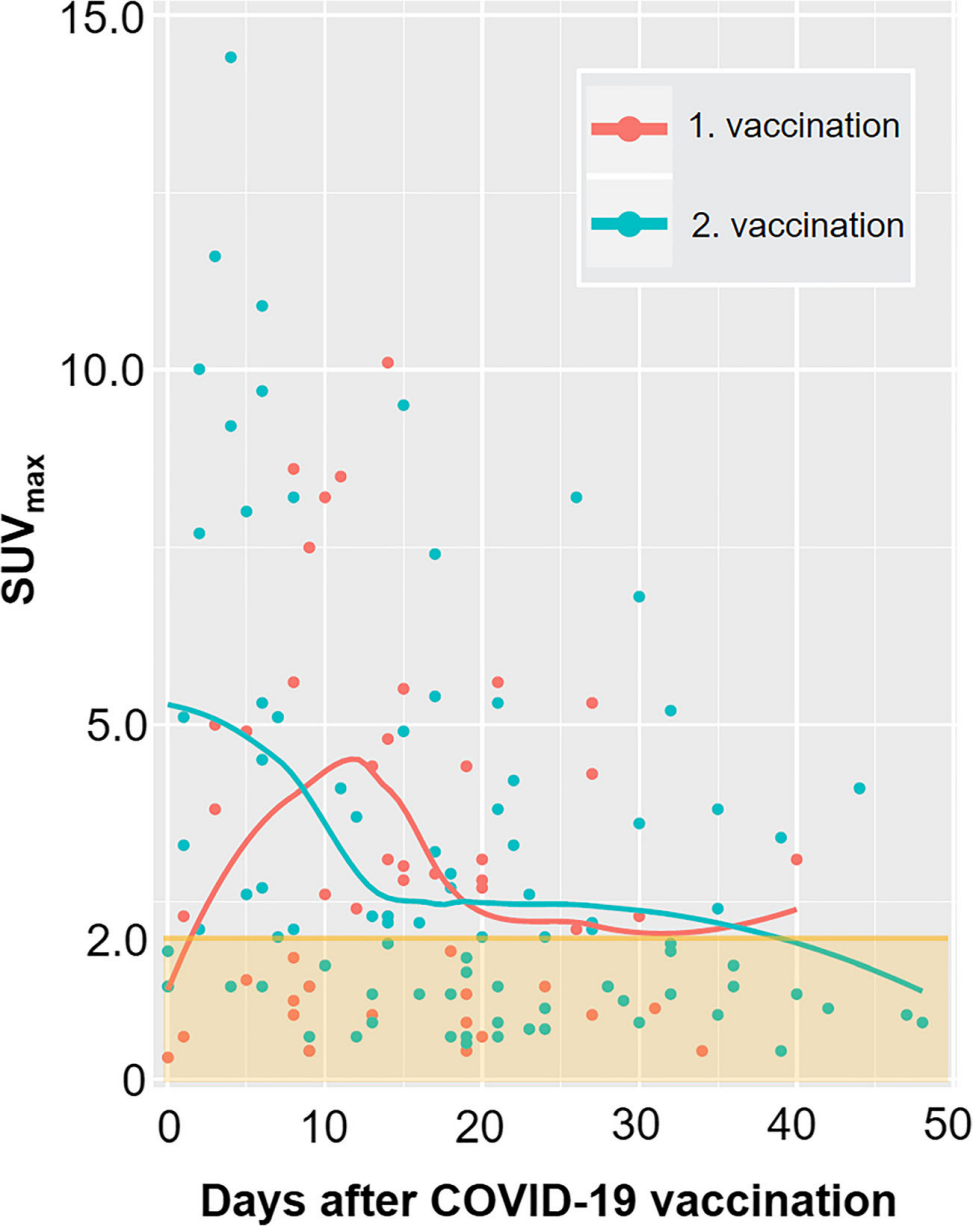
Scatter plot of maximum standardized uptake values (SUV_max_) of lymph nodes ipsilateral to COVID-19 vaccination and local polynomial regression fitting curves separated for first (red) and second (green) vaccination. Note: Based on qualitative and quantitative assessment (see Materials and Methods section), the lowest SUV_max_ in a patient deemed to have FDG-avid lymphadenopathy was 2.0 (orange line)

The mean FDG-uptake in the axillary lymph nodes was SUV_max_ 5.1 ± 3.4 (range 2.0 to 17.3) in 39/90 patients vaccinated with Pfizer-BioNTech and SUV_max_ 5.1 ± 2.7 (2.0 to 11.6) in 36/50 patients vaccinated with Moderna, *p* value = 0.542 (Table 3). Patients that received Moderna vaccines presented more frequently with FDG-avid lymphadenopathy (36/50; 72%) as compared to patients that received Pfizer-BioNTech (39/90; 43%), *p* value < 0.001, see Table 3. Patients receiving the second vaccination were significantly older, *p* value = 0.024, and received Pfizer-BioNTech more frequently, *p*-value < 0.001, Table 4. The single highest avidity was measured on the 14^th^ day after the first vaccination (SUV_max_ of 10.1) and on the 5^th^ day after the second vaccination (SUV_max_ of 17.3) (Fig. 1).

**Table 3:** Vaccine based analysis of patients’ characteristics and FDG PET/CT findings of study cohort (*n* = 140)

Characteristics	Pfizer-BioNTech (*n* = 90)	Moderna (*n* = 50)	*p* value^a^
Patient age, years	67 ± 13 (33–94)	67 ± 14 (25–91)	0.896
Sex, male	64 (71%)	37 (74%)	0.716
Number of vaccinations			< 0.001
1. vaccination	18 (20%)	30 (60%)	
2. vaccination	72 (80%)	20 (40%)	
Injection site			0.940
Right arm	18 (20%)	9 (18%)	
Left arm	72 (80%)	41 (82%)	
Day post vaccination, days	18 ± 13 (0–48)	16 ± 8 (0–40)	0.478
Patients with avid lymph nodes	39 (43%)	36 (72%)	0.001
SUV_max_ of avid lymph node	5.1 ± 3.4 (2.0–17.3)	5.1 ± 2.7 (2.1–11.6)	0.542

Values are means ± standard deviations (range), or frequencies (percentages)

^a^Wilcoxon test for paired non-parametric data and chi-squared-test for non-paired, non-parametric data

**Table 4:** Vaccination date-based analysis of patient characteristics and FDG PET/CT findings of study cohort (*n* = 140)

Characteristics	Post 1. vaccination (*n* = 48)	Post 2. vaccination (*n* = 92)	*p* value^a^
Patient age, years	64 ± 13 (33–91)	68 ± 13 (25–94)	0.024
Sex, male	35 (73%)	66 (72%)	0.883
Vaccine			< 0.001
Pfizer-BioNTech	18 (38%)	72 (78%)	
Moderna	30 (62%)	20 (22%)	
Injection site			0.859
Right arm	10 (21%)	18 (20%)	
Left arm	38 (79%)	74 (80%)	
Day post vaccination, days	15 ± 9 (0–40)	18 ± 12 (0–48)	0.268
Patients with avid lymph nodes	27 (56%)	48 (52%)	0.647
Pfizer-BioNTech	7 (39%)	32 (44%)	0.787
Moderna	20 (67%)	16 (80%)	0.682
SUV_max_ of avid lymph node	4.1 ± 1.7 (2.1–8.2)	5.4 ± 3.5 (2.0–17.3)	0.393

Values are means ± standard deviations (range), or frequencies (percentages)

^a^Wilcoxon test for paired non-parametric data and chi-squared-test for non-paired, non-parametric data

### Metastatic risk assessment and impact on clinical management

Assessing the metastatic risk of unilateral axillary lymphadenopathy based on oncological diagnosis and intensity of FDG uptake, we found FDG-avid lymph nodes (*n* = 75) *unlikely to* represent metastasis in 52/75 (69%; 37% of total study group) cases, *potentially* to represent metastasis in 15/75 (20%; 11% of total study group) cases, and *likely* to represent metastasis in 8/75 (11%; 6% of total study group) cases, respectively.

Reviewing the 75 patients with FDG uptake in the ipsilateral axillary lymph nodes, in a total of 17/140 (12%), we identified a potential impact on clinical management, mainly due to additional focused sonography of the region and fine-needle aspiration to exclude malignancy, as described in detail in Table 5. In these patients, the underlying etiologies were melanoma (12/17), breast cancer (1/17), pharyngeal cancer (1/17), paraneoplastic syndrome (1/17), chronic lymphocytic leukemia (1/17), and lymphoma (1/17).

**Table 5:** Cases (17/140) of patients where metabolically avid lymph adenopathy in the axilla ipsilateral to COVID-19 vaccination led to change of management

N	Age	Day p. vacci.	Vacc., site	Oncological diagnosis	SUV_max_	History, imaging finding	Impact on management recommendation
1^#^	75	8	M, left	Melanoma	8.6	Previously metastatic lymph nodes left cervical; new left axillary FDG-avid lymph node.	Additional sonography / fna
2^#^	69	13	M, left	Melanoma	4.4	Metastatic melanoma with unclear primary; metabolic active lymph node left axilla.	Additional sonography / fna
3^#^	55	14	M, left	Breast cancer	10.1	Operated right breast cancer with lymphadenectomy right axilla; new left axillary FDG-avid lymph node.	Additional sonography / fna
4^#^	49	15	M, left	Melanoma	5.5	Melanoma resected left neck; new FDG-avid left axillary lymph node.	Additional sonography / fna
5^#^	76	27	M, right	Melanoma	5.3	Melanoma resected left arm; new right axillary FDG-avid lymph node.	Additional sonography / fna
6^*^	77	2	M, left	Melanoma	10.0	Melanoma resected left neck; new left axillary FDG-avid lymph node.	Additional sonography / fna
7^*^	53	3	M, left	Melanoma	11.6	Melanoma resected left arm; new left axillary FDG-avid lymph node.	Earlier PET imaging restaging
8^*^	41	4	PfB, left	Paraneoplastic syndrome	14.4	Highly FDG-avid left axillary lymph node.	fna of lymph nodes
9^*^	33	5	PfB, left	Lymphoma	17.3	Unclear swelling of lymph nodes neck; highly FDG-avid left axillary lymph node.	fna of lymph nodes
10^*^	79	6	PfB, right	Melanoma	5.3	Melanoma resected left shoulder; new right axillary FDG-avid lymph node.	Additional sonography / fna
11^*^	77	6	PfB, left	Melanoma	10.9	Metastatic melanoma with unclear primary; FDG-avid left axillary lymph node.	Additional sonography / fna
12^*^	67	15	M, left	Pharyngeal cancer	9.5	Extensive pharyngeal cancer with lymph node metastasis level I - V cervical plus left axillary.	Earlier PET imaging restaging
13^*^	73	17	M, right	CLL	7.4	Mildly FDG-avid lymphadenopathy supra- and infradiaphragmal; imaging shows enhanced FDG-avidity in right axillary lymph nodes	Changing site of initially planned lymph node dissection
14^*^	64	23	M, right	Melanoma	2.6	Melanoma metastasis right chest wall; new right axillary FDG-avid lymph node.	Additional sonography / fna
15^*^	44	26	PfB, left	Melanoma	8.2	Metastatic melanoma; new left axillary FDG-avid lymph node.	Additional sonography / fna
16^*^	65	30	PfB, left	Melanoma	3.6	Melanoma resected right neck; new left axillary FDG-avid lymph node.	Additional sonography / fna
17^*^	75	39	PfB, right	Melanoma	3.4	Melanoma resected parietal right; new right axillary FDG-avid lymph node	Additional sonography / fna

*FDG*, fluorodeoxyglucose; *fna*, fine needle aspiration; *PET*, positron emission tomography; *vacci*., vaccination; *Vacc.*, vaccine (PfB = Pfizer-BioNTech; M = Moderna)

^#^Post 1. vaccination

^*^Post 2. vaccination

**Fig. 2:**
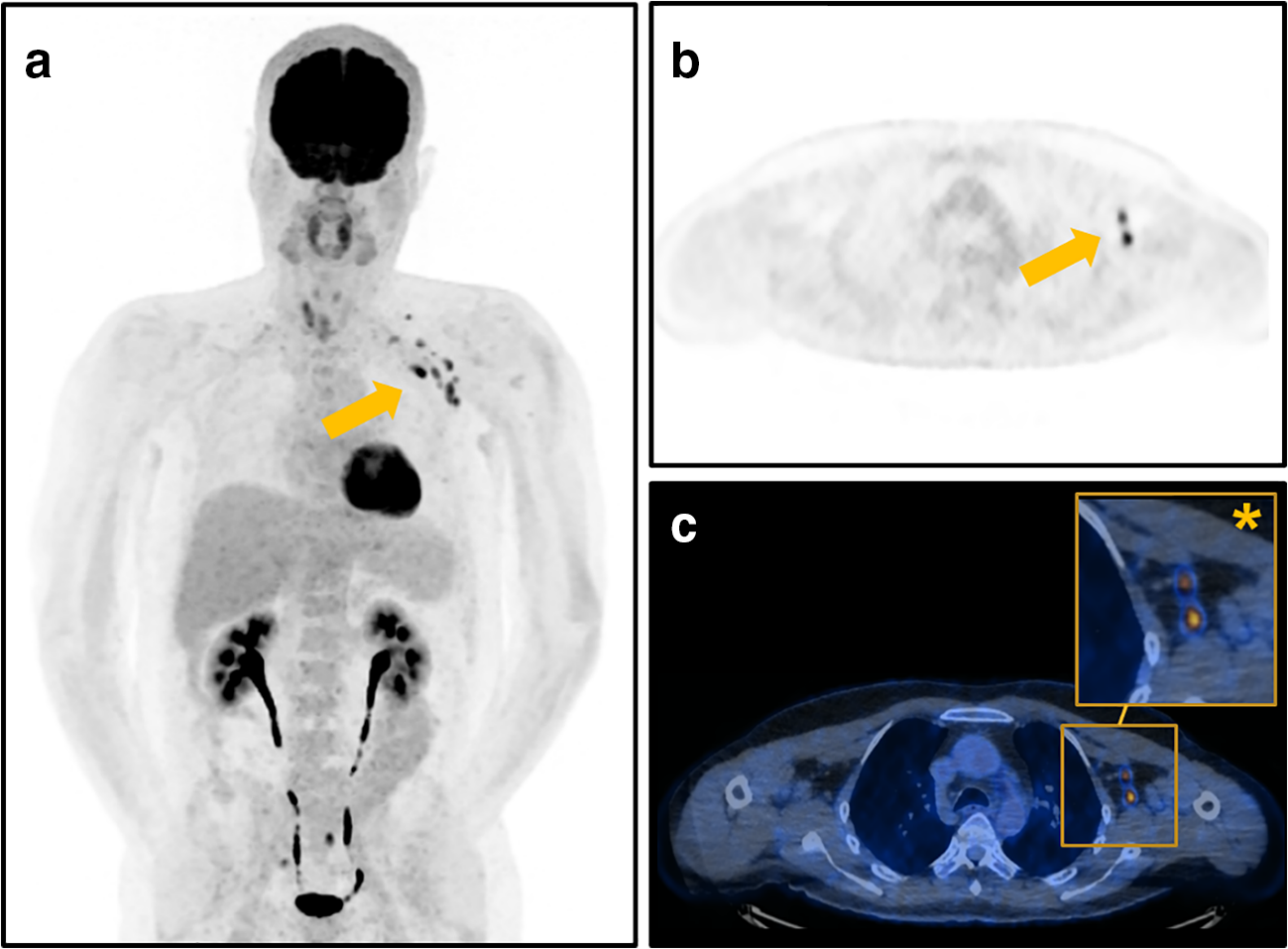
Representative images of a 53-year-old patient who underwent [^18^F]FDG PET/CT for restaging after resection of a melanoma of the left arm with FDG-avid axillary lymphadenopathy (arrows) after COVID-19 vaccination. The patient was scanned 3 days after the second vaccination with Moderna administered on the left side with SUV_max_ 11.6 of axillary lymph nodes. **a** Maximum intensity projection showing FDG-avid axillary lymph nodes, (**b**) axial PET image, and (**c**) fused PET/CT images with magnified image of the left axilla (asterisk). Due to the site of the primary lesion on the left arm and the FDG-avid lymph nodes, the patient was scheduled earlier for FDG PET/CT follow-up

A representative case of PET/CT with [^18^F]FDG-avid axillary lymphadenopathy is given in Fig. 2.

## Discussion

In this study, we sought to assess the overall frequency and intensity of [^18^F]FDG-PET/CT avid axillary lymph nodes ipsilateral to COVID-19 vaccination injection site, to further compare Pfizer-BioNTech and Moderna vaccines’ reactogenicity, as well as to assess potential clinical impact. One hundred and forty patients that were vaccinated with COVID-19 vaccines prior to undergoing [^18^F]FDG PET/CT for a variety of oncological indications were included.

The major findings of our study are as follows: First, 54% of patients presented with avid axillary lymph nodes ipsilateral to COVID-19 vaccination injection site. Second, FDG-avid axillary lymphadenopathy was most frequently seen on day 1–7 after vaccination (71% of patients) and showed a negative correlation with time after vaccination, but after 28 days and longer, still 38% of patients presented with FDG-avid lymph nodes. Third, the peak of lymph node activity tended to be earlier after the 2. vaccination as compared to the 1. vaccination. Fourth, patients vaccinated with Moderna presented with FDG-avid lymphadenopathy significantly more frequently compared to patients receiving Pfizer-BioNTech vaccines (i.e., 72% vs. 43%). Fifth, the FDG-avid lymph nodes had a potential impact on patient management (e.g., additional sonography/fine needle aspiration, earlier follow-up PET imaging) in 12% (17/140) of patients.

The diagnostic dilemma posed by lymphadenopathy after COVID-19 vaccination is highly dependent on the indication for FDG PET/CT. Oncological diseases that predominantly manifest in lymph nodes such as lymphoma; malignancies that tend to involve axillary, supraclavicular, or cervical lymph nodes, such as upper extremity or trunk melanoma, breast cancer, and head and neck cancer, as well as generally advanced-stage cancers, will be particularly prone to interpretive challenges. Indeed, in our cohort, we observed an impact on patient management most frequently in melanoma patients followed by breast cancer patients, head and neck cancer, and lymphoma. Various case reports have recognized the clinical implications resulting from FDG-avid lymph nodes as well, e.g., in breast cancer [12], melanoma [13, 14], and lung cancer [15], and all suggest history taking and raising awareness to consider the differential diagnosis of reactive lymphadenopathy.

Lymphadenopathy after COVID-19 vaccination was reported in 64 patients receiving the Pfizer-BioNTech vaccine compared to 6 patients in the placebo arm [16]. Further data from the US Center for Disease Control and Prevention (CDC) on local reactions after the Moderna COVID-19 vaccines reported that axillary swelling and tenderness was the second most common reported local reaction, after pain at site of injection. Among patients 18–64 years old, 12% receiving the vaccine reported axillary swelling and tenderness after the 1. vaccination, as compared to 5% in the placebo arm, and 16% after the 2. dose, compared to 4% in the placebo arm [3, 16]. This clinically detectable finding may only represent “the tip of the iceberg” as we found strikingly more patients with a reaction in the axillary lymph nodes on FDG PET/CT (up to 80% after 2^nd^ Moderna vaccination).

Both in vitro as well as clinical data suggest that the two mRNA vaccines investigated are more immunostimulatory and therefore inherently more immunogenic as compared to other traditional vaccine agents [17], which may potentially account for the more frequent and longer-lasting lymphadenopathy observed on imaging as compared to other agents (e.g., H1N1 with 29% [5] vs. 54% in our study).

A very recent study from Israel [9] reported high rates of FDG-avid axillary lymphadenopathy after vaccination with Pfizer-BioNTech (36% after the 1. dose, 54% after the 2. dose, overall 46%). This number is in line with the results from our study where we observed 39% of patients with avid lymph nodes after the 1. dose, 44% after the 2. dose and overall 43%, respectively. Interestingly, this number is well exceeded in patients that received Moderna vaccines with 67% after the 1. vaccination and even 80% after the 2. vaccination (overall 72%).

Our study has some limitations. First, the study group is relatively small, but this is reflected by the currently limited access to vaccination agents in our country. Second, as Moderna was introduced later in our country, we have more patients after the 1. vaccination in this group as compared to Pfizer-BioNTech which was mostly administered twice already; however, as we observed consistently higher rates of FDG-avid lymph nodes after Moderna vaccination after the 1. and 2. vaccinations, we feel that this does not affect this finding. Third, we were not able to obtain pathological proof of non-malignant (i.e., reactive) changes at the site of axillary lymphadenopathy, but by including only patients with unilateral active lymphadenopathy, we limited this potential bias. Fourth, we have not obtained follow-up imaging to assess how long the FDG-activity is persisting. Future studies may assess this topic as the vaccines are likely to remain in use for the coming time. Fifth, the retrospective assessment of metastatic risk in our study evaluated a scenario assuming no history of previous vaccination. Although this does not necessarily reflect factual recommendations and decisions of tumor boards, it provides a more accurate estimation of the frequency of diagnostic predicaments after COVD-19 vaccination.

In conclusion, metabolically active lymphadenopathy after COVID-19 vaccination is commonly seen in FDG PET/CT and the frequency may differ depending on the vaccine administered. This may lead to diagnostic and even therapeutic dilemmas. To tackle this dilemma, we recommend to explicitly ask patients for prior vaccination before undergoing PET/CT. To avoid potentially confounding FDG-uptake axillary lymph node, in oncological patients with lateralized upper body primary, the COVID-19 vaccines should be administered in the contralateral arm.

## Abbreviations

CT: Computed tomography
[^18^F]FDG: F18-Fluorodeoxyglucose
PET: Positron emission tomography
SUV_max_: Maximum standardized uptake value
VOI: Volume of interest

## Declarations

### Guarantor

The scientific guarantor of this publication is Dr. Michael Messerli.

### Statistics and biometry

No complex statistical methods were necessary for this paper.

### Methodology

• retrospective

• observational study

• performed at one institution
